# Contribution of influenza viruses to medically attended acute respiratory illnesses in children in high‐income countries: a meta‐analysis

**DOI:** 10.1111/irv.12400

**Published:** 2016-08-18

**Authors:** Sarah A. Buchan, Travis S. Hottes, Laura C. Rosella, Natasha S. Crowcroft, Dat Tran, Jeffrey C. Kwong

**Affiliations:** ^1^ Dalla Lana School of Public HealthUniversity of TorontoTorontoONCanada; ^2^ Public Health OntarioTorontoONCanada; ^3^ Institute for Clinical Evaluative SciencesTorontoONCanada; ^4^ Division of Infectious DiseasesDepartment of PediatricsThe Hospital for Sick ChildrenUniversity of TorontoTorontoONCanada; ^5^ Department of Family and Community MedicineUniversity of TorontoTorontoONCanada; ^6^ University Health NetworkTorontoONCanada

**Keywords:** acute respiratory illness, child, influenza, meta‐analysis, polymerase chain reaction

## Abstract

**Aim:**

The burden of disease in children attributable to influenza viruses is difficult to quantify given the similarity of symptoms caused by infection due to influenza and other viruses. This uncertainty impacts clinical decision‐making and estimates of burden. We aimed to systematically review the literature to determine the proportion of healthy children presenting for health care with an acute respiratory illness (ARI) who have laboratory‐confirmed seasonal influenza (PROSPERO ID#CRD42014013896).

**Method:**

We searched Ovid MEDLINE, EMBASE, Scopus, and references of included articles. We included studies that used polymerase chain reaction methods to test for influenza in healthy children aged ≤5 years who presented for health care in high‐income countries with an influenza‐like or ARI. A standardized form was used to collect data on positivity and other relevant study elements.

**Results:**

Seventeen studies covering 12 different influenza seasons were included. The proportion of influenza positivity ranged from 11% to 56%. Subgroup analyses were performed by influenza season, continent, healthcare setting, age group, and vaccination status. Higher influenza positivity was reported among children aged 3–5 years compared with children aged ≤2 years, and for unvaccinated children.

**Conclusion:**

The minority of healthy patients aged ≤5 years with medically attended influenza‐like or acute respiratory symptoms have laboratory‐confirmed influenza virus infection, although this varied by influenza season. Prevention efforts should be targeted accordingly.

**Statement:**

Most influenza‐like illnesses are not laboratory‐confirmed and have similar clinical presentations. Consequently, the true contribution of influenza to acute respiratory infections in children remains uncertain. Our systematic review estimates that this proportion ranges from 11% to 56%. This finding can help both clinicians and public health professionals target prevention.

## 1 Introduction

Annual epidemics of seasonal influenza continue to cause substantial morbidity and mortality among children.^1^, ^2^, ^3^ Previous studies have used non‐specific outcomes such as influenza‐like illness (ILI) or acute respiratory illness (ARI) to estimate disease burden and vaccine effectiveness.^4^ However, other respiratory viruses cause similar symptoms, making it challenging to distinguish infections due to influenza viruses from others.^5^ This uncertainty impacts clinical decision‐making and the estimation of the true burden of influenza, as well as vaccine effectiveness.^6^, ^7^ Laboratory testing identifies the specific virus responsible for the illness, but is not routinely performed in clinical practice; thus, the true contribution of influenza in the pediatric population remains uncertain.

While other reviews have examined the contribution of influenza viruses to burden in sub‐Saharan Africa or to acute lower respiratory infections (ALRI),^8^, ^9^ this study fills a gap in knowledge of the contribution to ILI/ARI in pediatric populations. To our knowledge, no other published systematic reviews have quantified the contribution of influenza viruses to medically attended respiratory illness in children in high‐income countries. The objective of this study was to systematically review the published peer‐reviewed literature evidence to determine the proportion of healthy children aged ≤5 years presenting for health care with ILI/ARI who have laboratory‐confirmed seasonal influenza.

## 2 Methods

### 2.1 Search strategy

We developed a detailed search strategy in consultation with a scientific librarian to identify articles related to children aged ≤5 years in high‐income countries (*population)* who had an ILI/ARI (*exposure)* and were tested for influenza with polymerase chain reaction (PCR) methods (*outcome)*. This strategy was applied to Ovid MEDLINE, EMBASE, and Scopus (from inception to August 6, 2014). Search terms included “influenza,” “flu,” “polymerase chain reaction,” “PCR,” “laboratory‐confirmed,” “child,” “infant,” and “adolescent.” Similar terms were combined with an “OR” operator and distinct terms linked with an “AND” operator. The full search strategy is outlined in File S1. Reference lists of included studies were also searched. The search strategy, along with the inclusion and exclusion criteria, was outlined in a registered study protocol (PROSPERO ID #CRD42014013896).^10^ The results of our systematic review and meta‐analysis are reported according to the Meta‐Analysis of Observational Studies in Epidemiology (MOOSE) and Preferred Reporting Items for Systematic Reviews and Meta‐Analyses (PRISMA) guidelines.^11^

### 2.2 Study selection

We included experimental, observational, and surveillance studies that used PCR methods to test for influenza infection in healthy children aged ≤5 years in high‐income countries who presented for health care with ILI/ARI, as defined by individual studies (see Table 1 for definitions). High‐income categorization was based on the definition developed by the World Bank.^12^ No language restrictions were placed on the initial review, although only full‐text articles available in English, French, or Spanish were included. We excluded studies in which PCR methods were not used or not used exclusively, studies that tested all participants (not just those with ILI/ARI), studies in which all participants were >5 years of age or which did not report results for this age group separately, studies of children with underlying medical conditions, studies that did not report each influenza season separately, studies that reported outbreak investigations, studies reporting interim estimates for an ongoing influenza season, studies that focused on diagnostic techniques, genetic characterization, clinical characteristics of patients, or describing the use of surveillance systems, studies that only reported on the 2009 A(H1N1) pandemic season, and studies of co‐infections that did not report influenza separately. Conference proceedings and abstracts, non‐peer‐reviewed reports, reviews, letters, editorials, case reports, and case series were excluded.

**Table 1:** Characteristics of included studies

Study author, publication year	Study design	Influenza season(s)	Study population	Country, continent	Included age range	Healthcare setting(s)	Case definition	Sample size (Total)
Andrews, 2014^25^	Test‐negative case–control	2012–2013	All ages	UK, Europe	≤5 y	Physician office	Acute respiratory illness with physician‐diagnosed fever or complaint of feverishness	294
Belongia, 2015^20^	Test‐negative case–control	2007–2008	All ages	USA, North America	≤5 y	Mix: Physician office, Hospital	Acute respiratory illness with symptoms of feverishness, chills, or cough	213
Castilla, 2013^26^	Test‐negative case–control	2011–2012	All ages	Spain, Europe	≤5 y	Mix: Physician office, Hospital	Sudden onset of any general symptom (fever or feverishness, malaise, headache, or myalgia) in addition to any respiratory symptom (cough, sore throat, or shortness of breath)	84
Chatzpolou, 2012^27^	Surveillance	2008–2009	Pediatric	Greece, Europe	≤2 y	Physician office	Not reported	42
Eisenberg, 2008^21^	Test‐negative case–control	2003–2004, 2004–2005	Pediatric	USA, North America	6–59 mo	Physician office	Presenting complaint of cough, earache, fever (identified through medical documentation or parent report), nasal congestion/runny nose, shortness of breath/rapid or shallow breathing, sore throat, vomiting after cough, or wheezing	2003–2004: 3622004–2005: 752
Fielding, 2011^33^	Test‐negative case–control	2007–2008, 2008–2009	All ages	Australia, Oceania	≤4 y	Physician office	History of fever, cough, and fatigue/malaise	2007–2008: 142008–2009: 9
Janjua, 2012^22^	Test‐negative case–control	2007–2008	All ages	Canada, North America	6 mo–2 y	Physician office	Acute onset of fever and cough and the presence of ≥1 of the following: sore throat, arthralgia, myalgia, or prostration	38
Jiminez‐Jorge, 2012^28^	Test‐negative case–control	2010–2011	All ages	Spain, Europe	≤4 y	Physician office	Sudden onset of symptoms, and at least one of these four systemic symptoms (fever or feverishness, malaise, headache, myalgia), and at least one of these three respiratory symptoms (cough, sore throat, shortness of breath), in the absence of other suspected clinical diagnosis	103
Kelly, 2011^34^	Test‐negative case–control	2008–2009	Pediatric	Australia, Oceania	6 mo–5 y	Physician office; Emergency Department	Documented fever with oral temperature >38°C, with at least one acute respiratory symptom or sign	289
Martinez‐Baz, 2013^29^	Test‐negative case–control	2010–2011	All ages	Spain, Europe	≤5 y	Mix: Physician office, Hospital	Sudden onset of any general symptom (fever or feverishness, malaise, headache, or myalgia) in addition to any respiratory symptom (cough, sore throat, or shortness of breath)	19
Pebody, 2013^30^	Test‐negative case–control	2010–2011	All ages	UK, Europe	≤5 y	Physician office	Acute respiratory illness with fever or complaint of feverishness	317
Rezza, 2006^31^	Surveillance	2004–2005	All ages	Italy, Europe	≤2 y	Physician office	Presence of fever >37.5°C and at least one other symptom (headache, malaise, myalgia, chills or sweats, retrosternal pain, asthenia) and one respiratory symptom (cough, sore throat, nasal congestion, or runny nose)	14
Staat, 2011^23^	Test‐negative case–control	2005–2006, 2006–2007	Pediatric	USA, North America	6–59 mo	Physician office; Hospital; Emergency Department	Not reported	2005–2006: 268 2006–2007: 260
Sung, 2009^36^	Surveillance	2005–2006	Pediatric	Hong Kong, Asia	≤5 y	Hospital	Sudden onset (<36 h) of one or more of the following symptoms and signs: rhinorrhoea, cough, sore throat, earache, hoarseness, stridor, wheeze, dyspnoea with or without fever	475
Treanor, 2012^24^	Test‐negative case–control	2010–2011	All ages	USA, North America	6 mo–2 y	Mix: Physician office, Emergency Department, Hospital	ARI with a duration of ≤7 d with documented fever or history of feverishness or cough	784
Turner, 2014^35^	Test‐negative case–control	2012–2013	All ages	New Zealand, Oceania	6 mo–5 y	Hospital	Severe acute respiratory infection defined as a patient requiring hospitalization with a history of a fever or a measured temperature ≥38°C and cough and onset within the past 7 d	334
Zambon, 2001^32^	Surveillance	1995–1996, 1996–1997, 1997–1998	All ages	UK, Europe	≤5 y	Physician office	Symptoms of fever, cough, and respiratory tract illness	1995–1996: 190 1996–1997: 108 1997–1998: 132

### 2.3 Review process

The titles and abstracts of retrieved articles were reviewed for general relevancy (SB). Studies meeting the predefined inclusion criteria were included for full‐text review, performed independently by two reviewers (SB and TSH). The inter‐rater reliability for studies selected for inclusion in the review was good (κ=0.79, 95% CI, 0.64–0.94), and any disagreements (n=7 studies) were resolved through discussion and consensus.

### 2.4 Data extraction

We used a standardized data extraction form to collect data on the following study elements: study publication details (author, journal, year of publication), study design, influenza season(s) and period of circulation, age groups, country of study, study setting (hospital, emergency department, physician office), outcome with case definition, number of participants studied, and number testing positive for influenza.

### 2.5 Outcome

The outcome of interest was the proportion of swabbed children testing positive for influenza after presenting for health care with ILI/ARI.

### 2.6 Assessment of risk of bias

We used a Newcastle–Ottawa scale adapted for cross‐sectional studies by Herzog et al.^13^ to assess the quality of included studies as they related to our outcome: the proportion tested who were influenza positive. Given the nature of our study question, we chose a tool that related to our outcome over one that assessed the quality of the study in general. This scale evaluated the quality of the study as it related to domains of selection, comparability, and outcome. We extracted a proportion from the studies that was not directly reported; therefore, the statistical test criterion in the outcome domain was deemed not applicable to this study.

### 2.7 Data synthesis and analysis

A stratified meta‐analysis was planned a priori due to anticipated heterogeneity of the estimates. Subgroup categories were as follows: influenza season, continent, age group (≤2 years, 3–5 years, or ≤5 years), study setting (hospital, emergency department, or physician office), and vaccination status (fully vaccinated, partially vaccinated, or unvaccinated). We analyzed strata with at least two eligible studies. A post hoc analysis was performed to examine positivity by timing of data collection, focusing on whether testing was restricted to periods when influenza viruses were in circulation, as defined by individual studies.

Meta‐analyses were performed in MetaXL (Version 2.0, EpiGear International Ltd, Queensland, Australia) with the inverse variance heterogeneity (IVhet) method.^14^ Unlike a random effects (RE) estimate, this method maintains the inverse variance weights of individual studies and provides a more conservative confidence interval.^14^, ^15^, ^16^ Given the high levels of heterogeneity and the wide range of sample sizes in this study, we chose the IVhet model for the primary analysis.^15^, ^16^, ^17^ We repeated the pooled analysis with the RE model using the DerSimonian and Laird method with double arcsine transformation to stabilize the variance,^15^ in order to compare the results. The analysis was run in R Statistical Software using the *meta* package to provide a prediction interval to estimate the range of values that future studies can be expected to fall within.^18^

Forest plots were created for each subgroup in order to examine clinical (e.g., age, setting, influenza season) and methodological (e.g., study design, study quality) heterogeneity. Statistical heterogeneity was assessed using the Cochran Q statistic (with *P*<.10 indicating statistical significance), as well as the *I* ^2^ statistic.^19^ We calculated the proportions (with corresponding 95% confidence intervals) of PCR‐confirmed influenza among children presenting with ILI/ARI overall and for each subgroup.

## 3 Results

**Figure 1:**
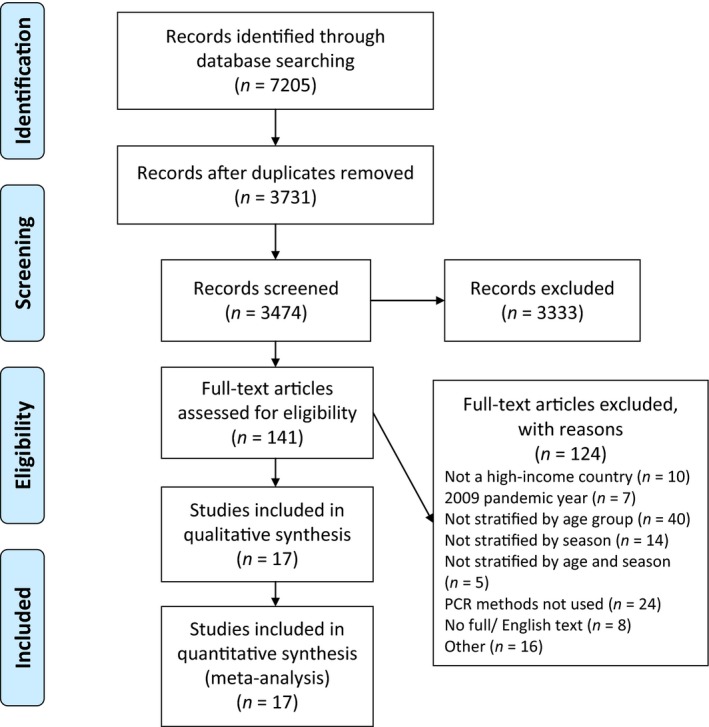
PRISMA flow diagram of study inclusion

A total of 3474 titles and abstracts were screened and 141 were selected for full review (Fig. 1). Of these, 17 studies satisfied the inclusion criteria and were included (Table 1).

The included studies presented data from 12 different influenza seasons between 1995 and 2012 with a total of 5101 participants (1042 cases of laboratory‐confirmed influenza). As some studies reported results from more than one influenza season, there were 22 entries across 12 influenza seasons. We identified five studies from North America,^20^, ^21^, ^22^, ^23^, ^24^ eight studies from Europe,^25^, ^26^, ^27^, ^28^, ^29^, ^30^, ^31^, ^32^ three studies from Oceania,^33^, ^34^, ^35^ and one study from Asia.^36^ Four studies reported outcomes for children aged ≤2 years of age only,^22^, ^24^, ^27^, ^31^ 11 studies for all children aged ≤5 years,^21^, ^25^, ^26^, ^28^, ^29^, ^30^, ^32^, ^33^, ^34^, ^35^, ^36^ and two studies reported results for two separate pediatric age groups.^20^, ^23^ One study reported results for children aged ≤2 years and 3–5 years separately,^20^ whereas the other study reported results for children aged ≤1 year and 2–5 years separately.^23^ For this review, the 2‐5 year age group was included in the older subgroup. The majority of studies took place in physician offices, with nine reporting on children presenting to this setting only.^21^, ^22^, ^25^, ^27^, ^28^, ^30^, ^31^, ^32^, ^33^ Two studies reported findings from children admitted to hospital,^35^, ^36^ four studies from children in a mix of settings,^20^, ^24^, ^26^, ^29^ and two studies reported results separately for more than one setting.^23^, ^34^ One study was conducted in all three settings, but only used PCR testing in the physician office setting; as such, only this setting was included in the analysis.^21^ Vaccination status was only presented by pediatric subgroups in four studies,^20^, ^21^, ^23^, ^34^ including one study that included vaccinated participants only.^20^ An additional study reported vaccination status, but had only one study participant and was therefore not included in the pooled estimate.^33^ Most studies tested specimens retrieved through nasopharyngeal or nasal swabs; however, four studies collected nasal and throat swabs.^21^, ^23^, ^24^, ^33^ One study collected only throat swabs^31^ and two studies did not report the type of specimen collected.^25^, ^28^ There were four surveillance studies,^27^, ^31^, ^32^, ^36^ while all others used the test‐negative case–control design. This approach is a modification to case–control studies, whereby patients seeking healthcare services with similar influenza‐like symptoms are swabbed; those testing positive for influenza become the cases, whereas those testing negative serve as controls. Eleven of the test‐negative case–control studies provided evidence that influenza was in circulation during the period of specimen collection,^20^, ^21^, ^22^, ^23^, ^24^, ^26^, ^29^, ^33^, ^34^, ^35^ while the remaining two studies did not explicitly state this.^28^, ^30^

In evaluating risk of bias using the modified Newcastle–Ottawa scale, we found that of the 17 included studies, five were at low risk (4–5 stars), 11 at moderate risk (2–3 stars), and one at high risk in the selection domain (Table S1 and Fig. S1). Few studies reported or compared non‐respondents or had adequate sample size for the included age group. In the comparability domain, four studies were at low risk (2 stars) and 13 studies at moderate risk (1 star). As this study was restricted to patients tested with PCR methods, all studies were at low risk in the outcome domain.

**Figure 2:**
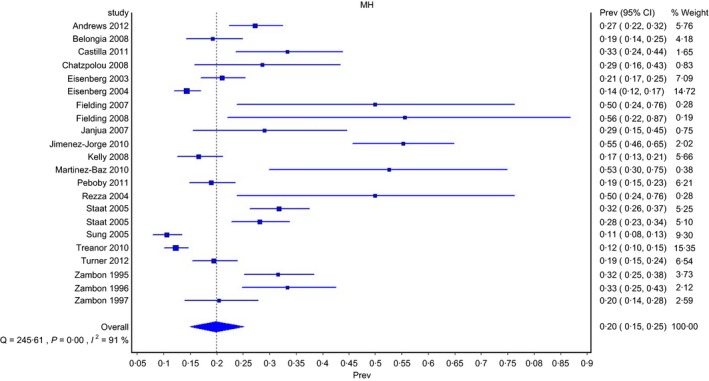
Forest plot of proportion of PCR‐confirmed influenza in children aged ≤5 years with an ILI or ARI using the inverse variance heterogeneity method, by study author and season

The pooled proportion across all studies indicated that 20% (95% CI, 15–25) of healthy children aged ≤5 years seeking health care with an ILI or ARI tested positive for influenza (Fig. 2). Influenza positivity ranged from 11% (95% CI, 8–13) to 56% (95% CI, 22–87) across the individual studies and from 14% (95% CI, 9–19) to 34% (95% CI, 27–41) across the various subgroups (Table 2, Fig. S2–S5). Influenza positivity was less than 35% in all but five estimates, which showed positivity of 50% or greater; however, four of these estimates were based on a sample of fewer than 20 children. Considerable heterogeneity was observed in the overall pooled proportion, based on the *I* ^2^ statistic (*I* ^2^=91%, 95% CI, 88–93) and Cochran Q test (χ^2^=245.6, *df*=21, *P*<.001); high levels of heterogeneity were also evident in most subgroups (Table 2). Influenza positivity remained unchanged when the study with high risk of bias in the selection domain was removed. No significant differences were seen when comparing positivity by study design. The pooled proportion calculated using the RE method was 26% (95% CI, 22–31), with a prediction interval of 8%–49%.

**Table 2:** Influenza positivity according to subgroup

	Subgroup	Number of seasons	Proportion positive (95% CI)	*I* ^2^ Statistic^a^	Cochran's *Q* a (degrees of freedom, *P* value)
Region	North America	7	18 (12, 25)	92%	χ^2^=75.6 (d.f.=6, *P*<.001)
	Europe	8	29 (21, 37)	86%	χ^2^=62.7 (d.f.=9, *P*<.001)
	Oceania	3	19 (10, 29)	77%	χ^2^=13.2 (d.f=3, *P*<.001)
Study population	Pediatric	5	18 (12, 25)	92%	χ^2^=77.2 (d.f.=6, *P*<.001)
	All ages	9	22 (14, 30)	91%	χ^2^=158.1 (d.f.=14, *P*<.001)
Age group	≤2 y	6	16 (7, 26)	84%	χ^2^=37.0 (d.f.=6, *P*<.001)
	2–5 or 3–5 y	3	34 (27, 41)	48%	χ^2^=3.82 (d.f.=2, *P*=.15)
	≤5 y	11	20 (14, 26)	92%	χ^2^=171.7 (d.f.=14, *P*<.001)
Healthcare setting	Hospital	3	15 (7, 24)	84%	χ^2^=18.5 (d.f.=3, *P*<.001)
	Emergency department	3	24 (9, 39)	87%	χ^2^=15.7 (d.f.=2, *P*<.001)
	Physician office	12	23 (16, 30)	88%	χ^2^=129.3 (d.f.=15, *P*<.001)
	Mix	3	16 (3, 31)	92%	χ^2^=37.2 (d.f.=3, *P*<.001)
Vaccination status	Fully vaccinated	5	14 (9, 19)	71%	χ^2^=17.5 (d.f.=5, *P*<.01)
	Partially vaccinated	4	16 (10, 22)	59%	χ^2^=7.33 (d.f.=3, *P*=.06)
	Unvaccinated	5	22 (12, 33)	95%	χ^2^=73.4 (d.f.=4, *P*<.001)

a The *I* ^2^ statistic is used to quantify the degree of heterogeneity within each subgroup and the Cochran *Q* statistic used to test for statistical significance of this heterogeneity.^19^

### 3.1 Influenza Season

The proportion of influenza tests positive ranged from 15% in 2004–2005 to 35% in 2007–2008. Seasons with only one corresponding study were not examined. Additionally, due to having very few studies in each subgroup, the precision of the estimates was low. However, positivity was correlated with overall severity of the season in North America, as reported by the U.S. Centers for Disease Control and Prevention; for example, the 2007–2008 influenza season was moderately severe,^37^ and the positivity rate was higher for that season than others. There was no difference in positivity before vs after the 2009 pandemic season. There was also no significant difference in positivity when examining the studies by whether they restricted sample collection to periods when influenza viruses were actively circulating. However, definitions of influenza season did vary among the studies.

### 3.2 Continent

The proportion of influenza positives ranged from 18% to 29% across continents, although Asia was not included as it was only represented by one study. North America had the lowest proportion positive (18%, 95% CI, 12–25) (n=5 studies) while the European region had the highest (29%, 95% CI, 21–37) (n=8 studies).

### 3.3 Age group

Of children aged ≤2 years with ILI/ARI, 16% tested positive for influenza (95% CI, 7–26) (n=7 studies). In the studies that did not stratify the ages further (n=15 studies), children aged ≤5 years had a proportion positive of 20 (14–26). Children in the older subgroup (2–5 or 3–5 years) had the highest proportion of influenza positives at 34% (95% CI, 27–41) (n=3 studies). Information was not available to look at infants (<12 months), including those not eligible for vaccination (<6 months), separately.

### 3.4 Healthcare setting

The proportion of influenza positives was 15% (95% CI, 7–24) in inpatient settings (n=3 studies), 24% (95% CI, 9–39) in emergency departments (n=2 studies), and 23% (95% CI, 16–30) in physician offices (n=11 studies). In the remaining studies that did not separate the results by setting, the proportion was 16% (95% CI, 3–31) (n=4 studies).

### 3.5 Vaccination status

One study restricted participants to those vaccinated,^20^ and only three other studies reported vaccination status for children aged ≤5 years.^21^, ^23^, ^34^ Methods of obtaining vaccination status varied across these studies, including use of immunization registries, vaccination cards, provider record, or contacting primary care or vaccine providers. The proportion of influenza positives was highest for unvaccinated children (22%, 95% CI, 12–33) (n=3 studies) and lowest for fully vaccinated children (14%, 95% CI, 9–19) (n=4 studies). The percent positivity for those partially vaccinated fell between these values (16%, 95% CI, 10–22) (n=2 studies). When those known to be unvaccinated were removed, the overall percentage positivity decreased from 20% (95% CI 15–25) to 18% (95% CI 13–23).

## 4 Discussion

The results of this systematic review and meta‐analysis suggest that seasonal influenza viruses contribute to approximately 20% of medically attended respiratory illnesses in healthy young children in high‐income countries, with individual study estimates ranging from 11% to 56%. However, there was significant heterogeneity in this pooled proportion and the estimate varied by influenza season, region, study population, age group, healthcare setting, and vaccination status.

Our study demonstrates that children aged 3–5 years had a higher proportion of influenza positivity than children ≤2 years of age, and the proportion for those children aged ≤5 years (i.e., not stratified further by age) fell between these two groups. While symptom severity and risk of hospitalization are greater for children aged ≤2 years,^5^, ^38^, ^39^, ^40^ including those <6 months of age who are not eligible for vaccination, the percentage positive is higher in older children aged 3–5 years. Preschool children have the highest transmission potential,^41^, ^42^ and their interactions in school and daycare settings may increase their risk of exposure to influenza, which may help to explain the higher rate of positivity for this age group.^43^ Additionally, a lower threshold of disease severity for younger children may result in seeking health care, therefore increasing the denominator in this age group, which could lead to lower overall positivity; however, this mechanism could not be evaluated.

We are not aware of any prior reviews published on this topic for children in high‐income countries. One meta‐analysis of respiratory infections found that globally in 2008, 13% of pediatric ALRI were attributable to influenza viruses; however, this study had no geographic limitations and considered ALRI as opposed to ILI/ARI.^9^ Another systematic review examined the burden of seasonal influenza, including some PCR‐confirmed outcomes, and found the percent of outpatient ARI patients that tested positive for influenza ranged between 1% and 25%, but the study included all ages and was limited to sub‐Saharan African countries.^8^ Finally, another review described the burden of seasonal influenza in children, but it was not performed systematically nor did it include meta‐analysis.^44^ Our study demonstrates consistent findings with this review in noting a higher burden of influenza in outpatient settings.

Our study provides a better understanding of pediatric influenza epidemiology by estimating the contribution of influenza viruses to medically attended respiratory illnesses in young children.^45^, ^46^ Having better data on the contribution of influenza to ILI in this population can help to estimate the risk of disease in different populations; inform immunization uptake,^47^ programs, and prevention strategies; evaluate treatment plans; and plan for seasonal epidemics and potential pandemics.^45^ The WHO has noted that this information can guide the allocation of health resources and the establishment of thresholds of disease severity.^46^ These estimates can also be used in mathematical models to predict influenza burden,^48^, ^49^, ^50^ cost‐effectiveness studies,^51^ and clinical decision‐making.

Clinical decision‐making can be informed by knowing the proportion of ILI/ARI caused by influenza viruses. The need for confirmation of the diagnosis of influenza, especially in children, has been highlighted given the other viruses in circulation and the potential beneficial impact of treatment.^52^ Timely administration of antiviral treatment to patients with confirmed influenza infection can reduce the duration of symptoms and prevent transmission to others.^52^, ^53^ Additionally, confirmation of the infectious agent will reduce overprescribing of unnecessary antimicrobials (both antibiotics and antivirals),^54^ which can lead to side effects,^55^ and which may contribute to increased antimicrobial resistance.^56^, ^57^ Prescription practices vary with respect to influenza season and ILIs; children are more likely to receive antibiotic prescriptions during influenza season than at other times of the year,^58^ and diagnoses of ILI correlate with antiviral prescription use.^59^ With the understanding that only a relatively minor portion of ILI/ARI is due to influenza viruses, incorporating testing into routine clinical decision‐making for children presenting with these symptoms may lead to more targeted and appropriate care. While our pooled estimate does not represent an individual's probability of testing positive for influenza, it does estimate the average contribution of influenza viruses to ILI/ARI over the course of a season. We also included the range of positivity estimates across subgroups in addition to the pooled result; these estimates may be useful for future studies in designing protocols or calculating sample sizes.^60^

In order to generate an appropriate pooled estimate of influenza positivity through meta‐analysis, the choice of meta‐analytic method was important. Ultimately, the IVhet method was chosen over the RE method given the high level of heterogeneity, although both were calculated for comparison and a prediction interval was also calculated using the RE method.^61^

Heterogeneity was expected between studies primarily because the outcome was an absolute measure of positivity. While the majority of studies had the primary purpose of estimating influenza vaccine effectiveness, we derived a direct estimate of positivity from the sample. As ratio measures are more stable across studies than absolute measures,^62^ our corresponding estimates of positivity would be expected to have higher heterogeneity. Heterogeneity in the estimate of influenza positivity was also expected due to myriad factors, including varying severity by influenza season and differences in study design, as well as differences in the population under study, including healthcare setting, age, geographic location, and vaccination status. As such, we performed stratified analyses based on these variables and calculated influenza positivity for each subgroup. Even after stratification, considerable heterogeneity remained for all but one subgroup and the estimates should be interpreted with this heterogeneity in mind. While these analyses attempted to explore some of the variability in influenza positivity, more work is required to understand the wide range of positivity in pediatric populations. Our decision to provide an overall estimate in the presence of heterogeneity may be debated, but in the absence of any comparable estimate, and acknowledging the myriad factors that affect influenza circulation, we consider it a worthwhile contribution to the literature and for future planning purposes. Nevertheless, this estimate should be interpreted with caution.

A strength of this study was the rigor of the methods applied at all stages, including both the search for articles and the meta‐analysis. We employed a broad search strategy in order to capture all relevant literature related to influenza detection in children with ILI/ARI. The study was strengthened by choosing PCR as the method of laboratory confirmation, due to its high sensitivity and specificity,^63^ as well as its improved detection over viral culture by 2%–13%.^64^ The study was also strengthened using the IVhet model, as opposed to the more commonly used RE model.^65^ While the pooled proportion of positivity reflected in the former was lower than the latter, it was not influenced by small studies with high rates of positivity that may not reflect the true contribution of influenza to respiratory illnesses.

This study also had some limitations, the first of which related to the search and selection process of the studies. The search included studies found through three databases, but did not include any gray literature. This limits our findings as surveillance data are often included in non‐peer‐reviewed reports, such as through the Public Health Agency of Canada's Flu Watch or Public Health Ontario's Influenza Bulletin, at the national and provincial levels, respectively; surveillance may also be reported through individual hospitals. These data are often reported throughout the season at weekly or monthly intervals and would have added to the study, but would be greatly limited by inconsistencies in testing criteria and the types of tests used. As well, only articles with full text available in English, French, and Spanish were reviewed, although very few studies were excluded based on language restrictions. Second, we observed high levels of heterogeneity and some studies had very small sample sizes. Removal of the studies with small sample sizes in the sensitivity analysis did not appreciably change the overall estimate of positivity. While subgroup analyses were performed to try to account for some of the heterogeneity, large amounts of residual heterogeneity remained and are worth exploring. Although our choice of the IVhet model was preferred over the RE model in providing a more conservative estimate, it does not resolve the issue of unexplained heterogeneity. Readers should interpret this pooled estimate with an understanding of unexplained variation and recognize that further efforts are needed to identify and understand these sources of heterogeneity. Third, very few studies provided information on vaccination status for children, which may explain some of the variability in influenza positivity. Many studies reported on the number of laboratory‐confirmed influenza positive individuals by age group, but failed to report the vaccination status by age group as well; instead, this vital information was reported as a percentage of the entire study population. Having weekly data reported for all studies would have facilitated understanding how influenza behaves over the course of the season, especially in relation to other circulating viruses. Such viral data would also be useful as indirect measures of influenza burden for certain mathematical models.^48^, ^49^ Additionally, while the test‐negative case–control design employed by most of the included studies reduces bias related to healthcare‐seeking behavior, the results of this review reflect only the subset of individuals who seek health care for their illness, and do not represent the total burden of influenza nor the range of severity.

## 5 Conclusion

This study quantified and investigated the proportion of medically attended acute respiratory illnesses attributable to influenza in pediatric populations in high‐income countries. While the positivity was fairly consistent across seasons and locations, the remaining variability should be investigated and the pooled estimate should be interpreted with caution. Although only a minority of acute respiratory illnesses are caused by influenza viruses, influenza is still an important contributor to morbidity given the substantial number of respiratory illnesses in the pediatric population. This burden of disease can be reduced through seasonal influenza vaccination and other prevention strategies to reduce the risk of all respiratory viruses.

## Funding Source

Sarah Buchan's doctoral training is supported by a Public Health Agency of Canada and Canadian Institutes of Health Research Influenza Research Network (PCIRN) Trainee Award.

## Conflict of Interest

None to disclose.
