# Supplementary material for: Contribution of influenza viruses to medically attended acute respiratory illnesses in children in high‐income countries: a meta‐analysis
Source: Influenza Other Respir Viruses. 2016 Aug 18;10(6):444–54. doi: 10.1111/irv.12400 (PMC5059948; doi:10.1111/irv.12400)
Supplement: Supplementary file 1 [file IRV-10-444-s001.docx]

Figure S1: Quality assessment of included studies by domain, using a modified Newcastle-Ottawa scale for cross-sectional studies
