# Supplementary figures and images for: Contribution of influenza viruses to medically attended acute respiratory illnesses in children in high‐income countries: a meta‐analysis
Source: Influenza Other Respir Viruses. 2016 Aug 18;10(6):444–54. doi: 10.1111/irv.12400 (PMC5059948; doi:10.1111/irv.12400)

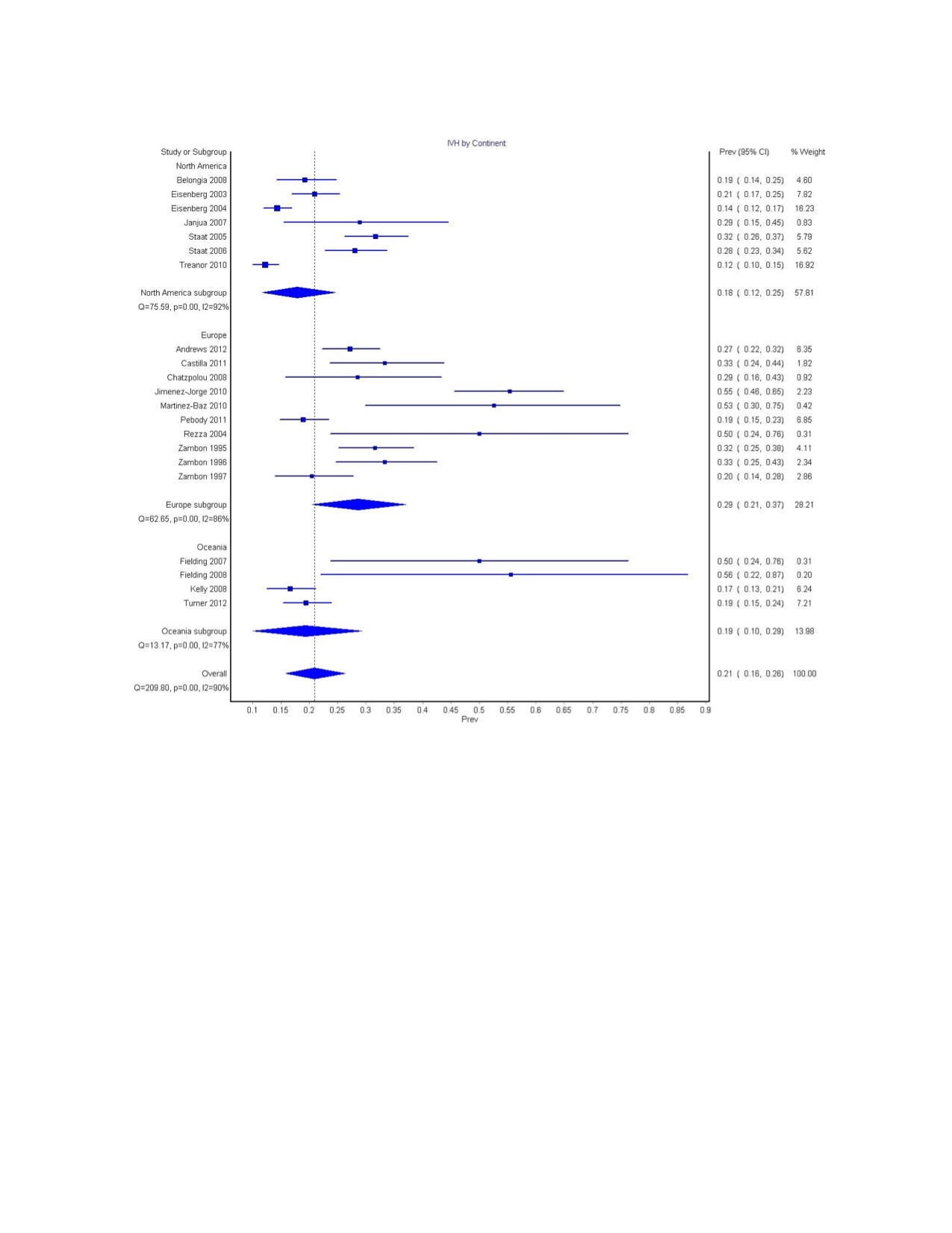

Supplement: Supplementary file 2 [file IRV-10-444-s002.tiff]

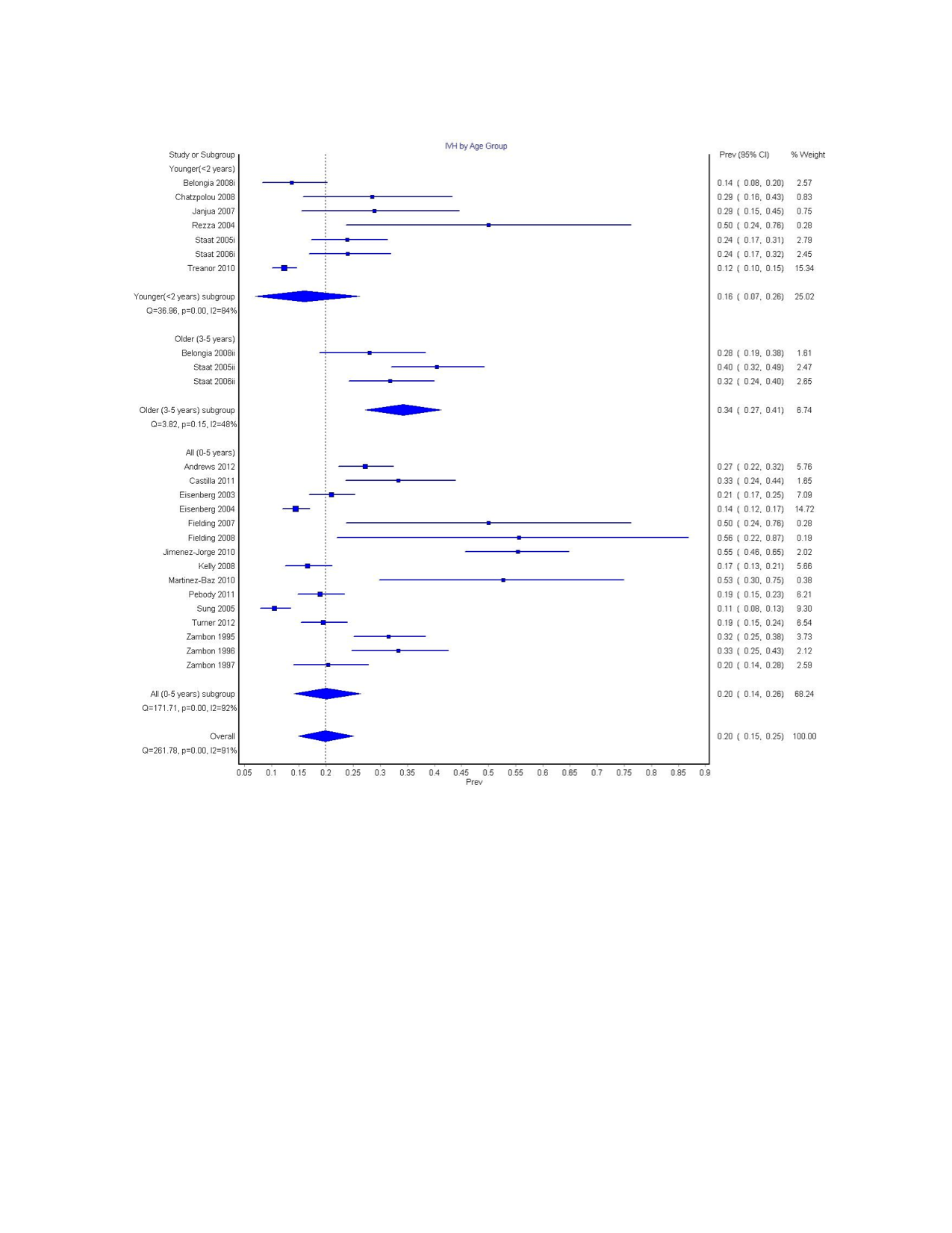

Supplement: Supplementary file 3 [file IRV-10-444-s003.tiff]

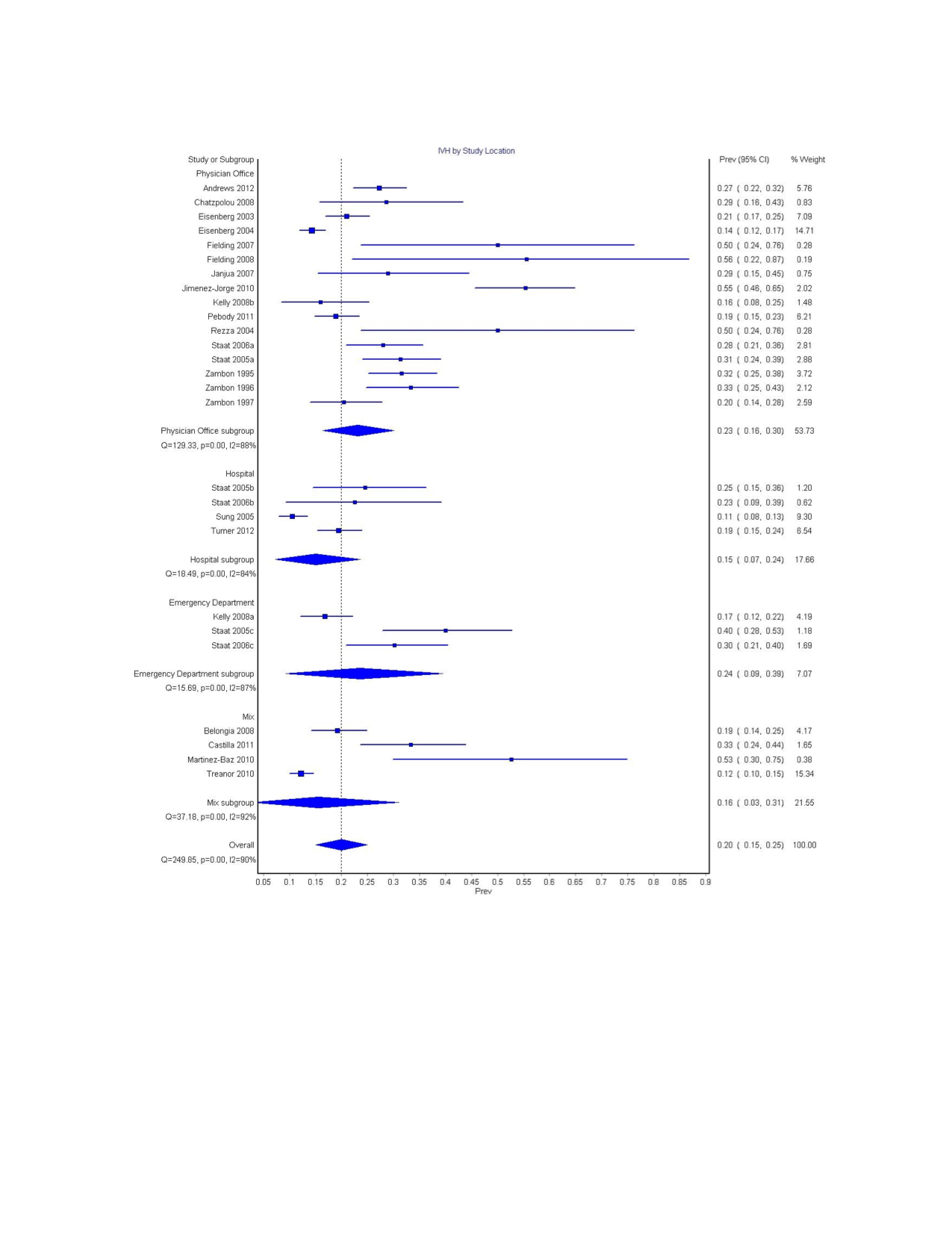

Supplement: Supplementary file 4 [file IRV-10-444-s004.tiff]

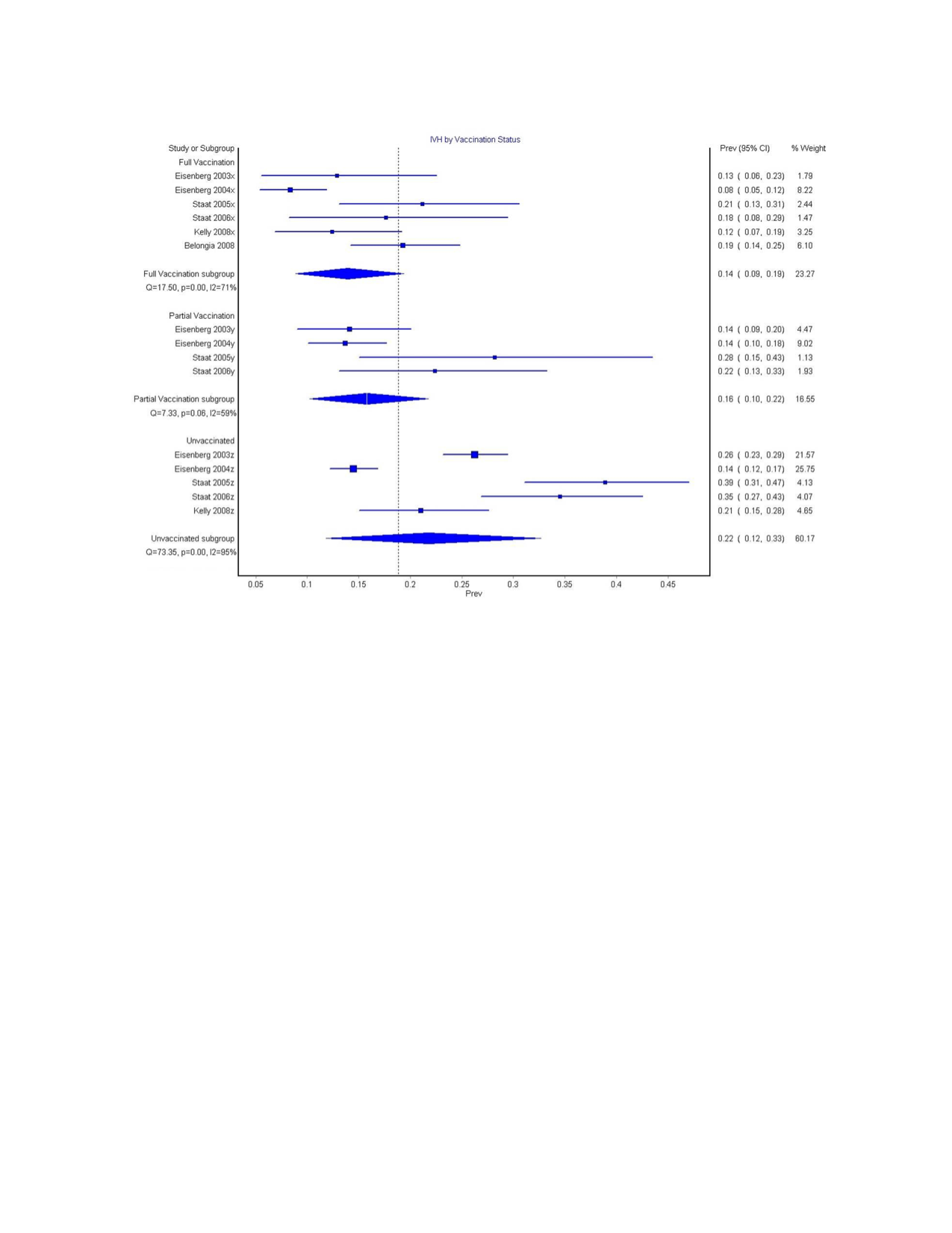

Supplement: Supplementary file 5 [file IRV-10-444-s005.tiff]
