# Supplementary material for: Contribution of influenza viruses to medically attended acute respiratory illnesses in children in high‐income countries: a meta‐analysis
Source: Influenza Other Respir Viruses. 2016 Aug 18;10(6):444–54. doi: 10.1111/irv.12400 (PMC5059948; doi:10.1111/irv.12400)
Supplement: Supplementary file 6 [file IRV-10-444-s006.docx]

Table S1: Quality assessment using a modified Newcastle-Ottawa Scale for cross-sectional studies

|  | Selection /5 | | | | Comparability /2 | Outcome /2 | |
| --- | --- | --- | --- | --- | --- | --- | --- |
| Author, Publication year | Representativeness of sample | Sample size | Non-respondents | Ascertainment of exposure | Subjects comparable | Assessment of the outcome | Statistical test |
| Andrews, 2014 | * | * |  | ** | * | ** | n/a |
| Belongia, 2014 | * |  |  | ** | * | ** | n/a |
| Castilla, 2013 | * |  |  | ** | * | ** | n/a |
| Chatzopoulou, 2012 |  |  |  | NR | * | ** | n/a |
| Eisenberg, 2008 |  | * | * | ** | ** | ** | n/a |
| Fielding, 2011 | * |  |  | ** | * | ** | n/a |
| Janjua, 2012 | * |  |  | ** | ** | ** | n/a |
| Jiminez-Jorge, 2012 |  |  |  | ** | * | ** | n/a |
| Kelly, 2011 | * | * |  | ** | ** | ** | n/a |
| Martinez-Baz, 2014 | * |  |  | ** | * | ** | n/a |
| Pebody, 2013 | * | * |  | ** | * | ** | n/a |
| Rezza, 2006 | * |  |  | ** | * | ** | n/a |
| Staat, 2011 |  | * | * | NR | ** | ** | n/a |
| Sung, 2009 |  | * |  | ** | * | ** | n/a |
| Treanor, 2012 | * | * |  | ** | * | ** | n/a |
| Turner, 2014 | Unclear | * |  | ** | * | ** | n/a |
| Zambon, 2001 | * |  |  | ** | * | ** | n/a |

NR: Not Reported
