# Supplementary material for: Contribution of influenza viruses to medically attended acute respiratory illnesses in children in high‐income countries: a meta‐analysis
Source: Influenza Other Respir Viruses. 2016 Aug 18;10(6):444–54. doi: 10.1111/irv.12400 (PMC5059948; doi:10.1111/irv.12400)
Supplement: Supplementary file 7 [file IRV-10-444-s007.docx]

## File S1: Complete Search Strategy

**Medline:**

| #1 | exp Influenza, Human/ OR exp Influenza Vaccines/ OR (influenza or flu).mp |
| --- | --- |
| #2 | exp Polymerase Chain Reaction/ OR polymerase chain reaction.mp OR (laboratory confirmed or lab confirmed).mp |
| #3 | # 1 and # 2 |
| #4 | (child: or adolescent or infan:).mp. |
| #5 | #3 and #4 |

**Embase:**

| #1 | 'influenza'/exp or 'Influenza virus'/exp or 'influenza vaccine'/exp or influenza.mp. or flu.mp |
| --- | --- |
| #2 | exp Polymerase Chain Reaction/ OR polymerase chain reaction.mp OR (laboratory confirmed or lab confirmed).mp |
| #3 | # 1 and # 2 |
| #4 | (child: or adolescent or infan:).mp. |
| #5 | # 3 and # 4 |

**Scopus:**

| #1 | Influenza.mp OR flu.mp |
| --- | --- |
| #2 | polymerase chain reaction.mp OR PCR.mp OR (laboratory confirmed or lab confirmed).mp |
| #3 | # 1 and # 2 |
| #4 | (child: or adolescent or infan:).mp. |
| #5 | # 3 and # 4 |
